# Cancer Incidence following Expansion of HIV Treatment in Botswana

**DOI:** 10.1371/journal.pone.0135602

**Published:** 2015-08-12

**Authors:** Scott Dryden-Peterson, Heluf Medhin, Malebogo Kebabonye-Pusoentsi, George R. Seage, Gita Suneja, Mukendi K. A. Kayembe, Mompati Mmalane, Timothy Rebbeck, Jennifer R. Rider, Myron Essex, Shahin Lockman

**Affiliations:** 1 Department of Medicine, Division of Infectious Diseases, Brigham and Women’s Hospital, Boston, Massachusetts, United States of America; 2 Botswana Harvard AIDS Institute Partnership, Gaborone, Botswana; 3 Department of Immunology and Infectious Diseases, Harvard School T.H Chan School of Public Health, Boston, Massachusetts, United States of America; 4 Department of Medicine, Harvard Medical School, Boston, Massachusetts, United States of America; 5 Department of Public Health, Botswana Ministry of Health, Gaborone, Botswana; 6 Department of Epidemiology, Harvard T.H. Chan School of Public Health, Boston, Massachusetts, United States of America; 7 Department of Radiation Oncology, University of Utah School of Medicine, Salt Lake City, Utah, United States of America; 8 National Health Laboratory, Botswana Ministry of Health, Gaborone, Botswana; 9 Department of Epidemiology, University of Pennsylvania, Philadelphia, Pennsylvania, United States of America; University of Washington, UNITED STATES

## Abstract

**Background:**

The expansion of combination antiretroviral treatment (ART) in southern Africa has dramatically reduced mortality due to AIDS-related infections, but the impact of ART on cancer incidence in the region is unknown. We sought to describe trends in cancer incidence in Botswana during implementation of the first public ART program in Africa.

**Methods:**

We included 8479 incident cases from the Botswana National Cancer Registry during a period of significant ART expansion in Botswana, 2003–2008, when ART coverage increased from 7.3% to 82.3%. We fit Poisson models of age-adjusted cancer incidence and counts in the total population, and in an inverse probability weighted population with known HIV status, over time and estimated ART coverage.

**Findings:**

During this period 61.6% of cancers were diagnosed in HIV-infected individuals and 45.4% of all cancers in men and 36.4% of all cancers in women were attributable to HIV. Age-adjusted cancer incidence decreased in the HIV infected population by 8.3% per year (95% CI -14.1 to -2.1%). However, with a progressively larger and older HIV population the annual number of cancers diagnosed remained constant (0.0% annually, 95% CI -4.3 to +4.6%). In the overall population, incidence of Kaposi’s sarcoma decreased (4.6% annually, 95% CI -6.9 to -2.2), but incidence of non-Hodgkin lymphoma (+11.5% annually, 95% CI +6.3 to +17.0%) and HPV-associated cancers increased (+3.9% annually, 95% CI +1.4 to +6.5%). Age-adjusted cancer incidence among individuals without HIV increased 7.5% per year (95% CI +1.4 to +15.2%).

**Interpretation:**

Expansion of ART in Botswana was associated with decreased age-specific cancer risk. However, an expanding and aging population contributed to continued high numbers of incident cancers in the HIV population. Increased capacity for early detection and treatment of HIV-associated cancer needs to be a new priority for programs in Africa.

## Introduction

Cancers now account for nearly a quarter of deaths among HIV-infected individuals, and will soon surpass AIDS illnesses as the leading cause of death of HIV-infected persons in high-income countries.[1] While the incidence of some AIDS-defining cancers (ADC) (Kaposi’s sarcoma [KS] and non-Hodgkin’s lymphomas [NHL]) has dropped dramatically with the use of combination antiretroviral therapy (ART),[2, 3] the risk of many non-AIDS-defining cancers (nADC) has increased.[4] In sub-Saharan Africa, where more than two-thirds of all HIV infections occur, there is limited knowledge of trends in cancer incidence. Reduced KS risk has been observed among ART recipients in sub-Saharan Africa,[5] but trends of population incidence of KS during periods of ART expansion have varied with decreased incidence in urban Uganda,[6] no significant change in rural sites in Uganda and Kenya,[5] and increased incidence in Malawi.[7] Incidence of NHL increased with available ART in urban Uganda[6] and remained unchanged in Malawi.[7] Understanding the impact of ART on cancer incidence in low and middle-income countries (LMICs) that bear the greatest burden of both HIV and cancer deaths[8] is of great importance.

Botswana has one of the most intense HIV epidemics with nearly one quarter of all adults infected,[9] but also one of the most robust ART programs. Botswana launched the first nationwide public ART program in Africa in 2002, and ART coverage exceeded 80 percent by 2008 for patients with CD4 ≤ 200 cells/μL, according to government estimates.[10] During this period, visits to the public oncology clinic in Botswana increased 3-fold[11] and wards previously used as overflow for AIDS infections were transitioned to oncology care. Utilizing records of individual cases from Botswana National Cancer Registry, we sought to evaluate trends in ADC and nADC incidence in the Botswana National Cancer Registry between 2003 and 2008 in the context of rapid expansion of ART in Botswana. The experience in Botswana may be indicative of upcoming trends in the region, where ART became available later and coverage is increasing.

## Methods

### Estimates of HIV Infected Population and Treatment

The Botswana HIV/AIDS Impact Survey, a nationally representative survey conducted by the government of Botswana, was performed in 2004[12] and 2008[13] and included serologic testing for HIV. Peak prevalence occurred among individuals aged 30 to 34 in 2004 (40.0%) and among individuals aged 40 to 44 in 2008 (41.3%) in 2008. These surveys, with linear extrapolation between measurements to permit age structure of the HIV infected population to change quarterly, were used to estimate HIV prevalence in 5-year age categories (collapsed for <20 and for >64 years of age due to sparse data). Age structure for the total population was estimated from the 2006 national census, and was assumed to stable during the study period.[14]

**Fig 1:**
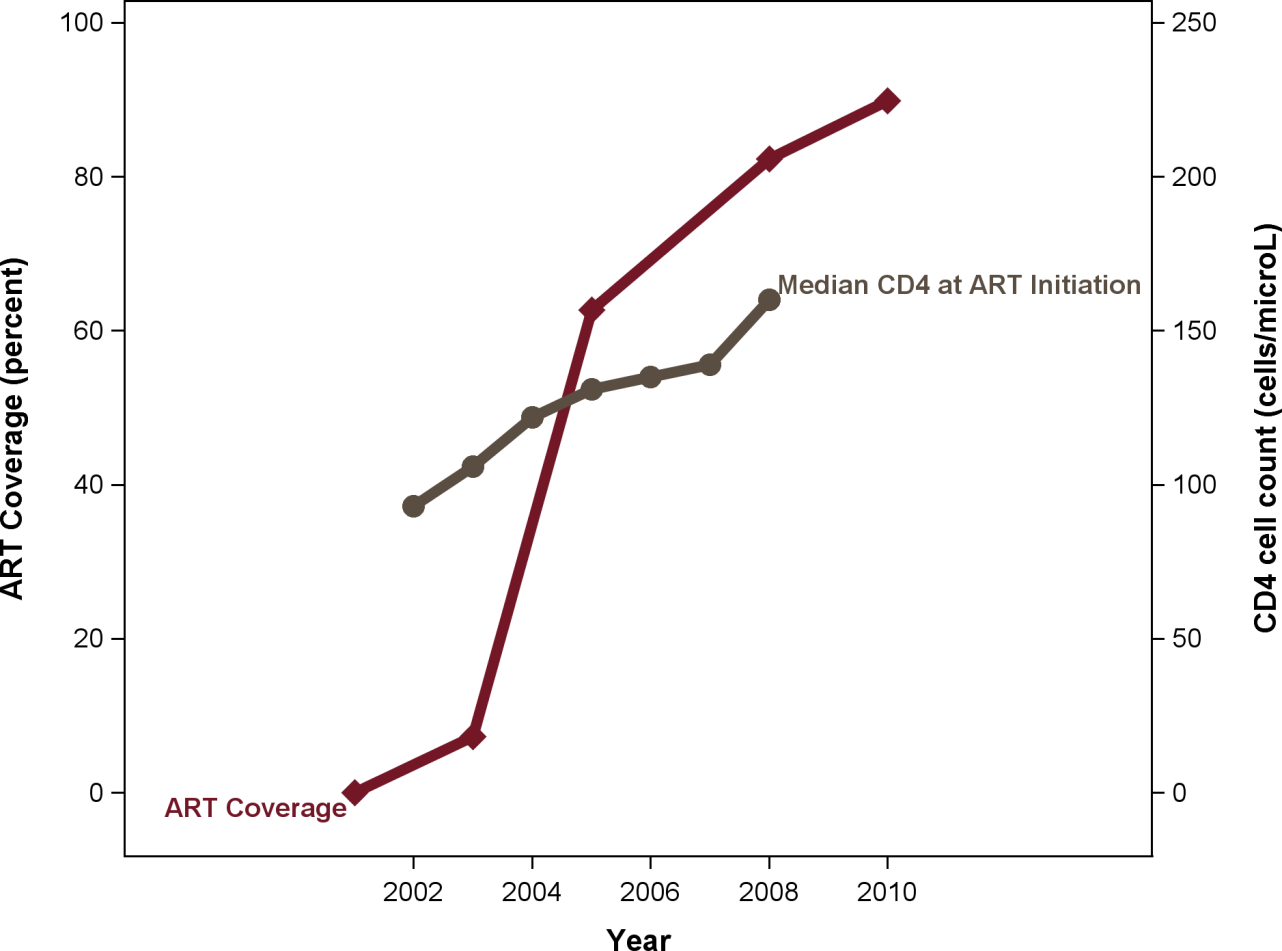
ART treatment coverage and median CD4 at ART initiation during the study period. Note: ART, combination antiretroviral therapy.

Initiated in 2002 in several urban centers, the ART coverage began to expand dramatically as the program started to decentralize in 2004 with consequent increases in median CD4 cell count at ART initiation (Fig 1). During the period included in this analysis (2003–2008), HIV-infected individuals with AIDS (WHO stage 3 or 4 HIV illness and/or CD4 ≤ 200 cells/μL) were ART-eligible. Standard first-line treatment during the period included co-formulated zidovudine and lamivudine with either efavirenz or nevirapine.[15, 16] Linear extrapolation from government estimates[10] of the proportion of treatment eligible individuals receiving ART in 2003, 2005, and 2008 were used to obtain quarterly ART coverage estimates used in regression models.

### Cancer Screening, Diagnosis, and Treatment

Cancer screening was not routinely available in the public sector during the study period. Programs supporting Papanicolaou testing were launched, but limitations of diagnostic capacity reduced the number of women effectively screened.[17] Consequently, with rare exception for some patients screened in the private sector, patients presented due to symptoms. Diagnostic procedures were generally performed at referral hospitals where oncologic care was provided.[18]

### Botswana National Cancer Registry

The Botswana National Cancer Registry (BNCR) of the Ministry of Health has registered cancer cases through active case surveillance throughout the country since 2003. Electronic and paper-based pathology, outpatient, and inpatient records and registers are reviewed at each facility. Both private and public facilities are included in the surveillance. Surveillance is focused at referral health facilities and pathology laboratories, and cases of cancer not in need of dedicated oncologic care may be underrepresented (e.g. limited Kaposi’s sarcoma treated with ART alone). There were no substantive changes in case ascertainment procedures employed by BNCR staff since 2003. The BNCR does not yet link to HIV registries and many records lack this information. During an assessment in 2013, a team from the International Agency for Research on Cancer estimated that the BNCR captured more than 85% of cancers in Botswana.[19]

### Analytic Methods

Consistency of cancer case capture by the BNCR during the study period was assessed using described semi-quantitative methods,[20, 21] including comparing the incidence of childhood cancers with regional incidence estimates,[22] evaluating longitudinal trends for selected common cancers requiring different diagnostic procedures and unlikely to be affected by expansion of ART (breast, colorectal, melanoma, and prostate cancer), and examining trends in the proportions of cases morphologically verified.

Registered cases were classified according to topography (ICD-10) and morphology (ICD-O-3). Squamous cell carcinomas arising in the larynx, hypopharynx, pharynx, oropharynx, and oral cavity were grouped as head and neck cancers. Squamous cell cancers involving the penis, anus, rectum, vulva, and vagina, but not the cervix, were considered as other anogenital cancers. Kaposi’s sarcoma, all non-Hodgkin’s lymphomas, and cervical cancer were considered AIDS-defining cancers (ADC) and all other cancers considered non-AIDS-defining (nADC).

To evaluate the impact of the initial rollout of ART in Botswana, cases reported to the registry during 2003 to 2008 were included in the analysis. Incidence was sex- and age-standardized to the World standard population.[23] Trends in incidence were assessed using overdispersed Poisson models including either calendar time or population ART coverage.

As HIV status was only available in the BNCR for a potentially non-representative subset of cases, we used inverse probability weighting methods [24] to adjust for selection bias and enable modeling by recorded HIV status. Briefly cases with known HIV status were weighted to account for comparable cases with unknown HIV status. We used the inverse of the probability of the case having a non-censored HIV status—conditional on calendar year, age, sex, cancer type, reporting facility, and region of patient residence—as the weights. Following the approach of Naimi *et*. *al*., weights were stabilized by including the probability of non-censoring conditional on quantiles of diagnosis date in the numerator.[25] Assuming exchangeability of cases with censored and non-censored HIV status conditional on included predictors, the resulting weighted population provides an unbiased estimate of the total population had HIV status been known for all cases. The magnitude of possible residual selection bias was assessed comparing model-predicted HIV prevalence and observed prevalence from a cohort enrolled at Princess Marina Hospital, [26] the largest oncology facility accounting for approximately 65% of nationally registered cases.

Incidence trends were modeled separately in the overall population, irrespective of HIV status, and by HIV status in the IPW population. Parametric approaches were used in the overall population. In order to account for increased variance due to weighting, significance testing and confidence intervals in the IPW population were estimated using observations from 1000 bootstrap samples. A strong correlation was found between calendar time and ART coverage (r^2^ = 0.87) during the study period; therefore, collinearity prevented modeling both factors simultaneously. Statistical analyses were performed with the use of the SAS statistical package, version 9.4 (SAS Institute, Cary, North Carolina). All tests were two-tailed and P-values of less than 0.05 were considered statistically significant.

The study was approved with a waiver of informed consent to use de-identified registry records by the institutional review boards of the Botswana Ministry of Health and the Harvard T.H Chan School of Public Health.

## Results

### Cancer Cases

From 2003 through 2008, 8479 new diagnoses of cancer were recorded, with 4615 (54.4%) cancer cases in women and 3864 (45.6%) in men. The median age at time of cancer diagnosis was 47 years for women and 50 for men. The majority of diagnoses were pathologically confirmed by histology (69.7%) or by cytological or hematologic examination (8.5%). Clinical or radiographic examination alone was used to diagnose 1138 (13.4%) cancers, nearly all of which were Kaposi’s sarcoma (98.7%). A total of 660 cases (7.8%) were identified only through review of death certificates. HIV status was recorded for 2438 (71.9%) cases of ADC and 1057 (20.8%) of nADC. Among cases with recorded HIV status, 92.6% of ADC and 52.0% nADC were in HIV-infected individuals.

### Consistency of Case Capture

We did not detect significant trends in recorded incidence for indicator cancers felt *a priori* to be unlikely to be affected by expansion of ART—breast (-1.4% annual, P = 0.38), colorectal (+1.0% annual, P = 0.76), melanoma (-4.6% annual, P = 0.29), prostate (-3.7% annual, P = 0.24). Considered together to increase power to observe smaller magnitude trends, no significant change (-1.7% annual, P = 0.18) was observed in the incidence of these indicator cancers. However, for all cancers there was a significant decline in the proportion of cancers identified by death certificates only, 13.1% during the first half of the study period compared with 2.5% during the second half (P<0.001). While this may represent increased access to histology and decreased intensity of review of death certificates, the decline in death certificate-only cases may also represent a decline in AIDS mortality during the study period. More than half of the cases diagnosed by death certificate only were from individuals who died of AIDS and had a cancer diagnosis (most commonly Kaposi’s sarcoma) noted on their death certificate. The incidence of cancer in children under the age of 15–6.2 for girls and 7.5 for boys (per 100,000)—was within published ranges for sub-Saharan Africa, but in the bottom decile for registries worldwide.[20] HIV prevalence was similar in the IPW population in 2008, 57.2% (95% CI 55.6 to 58.9), and enrollees in the Princess Marina Hospital cohort enrolled from 2010 to 2012, 59.4% (95% 50.1 to 67.6%).[26]

### Overall Cancer Incidence

**Fig 2:**
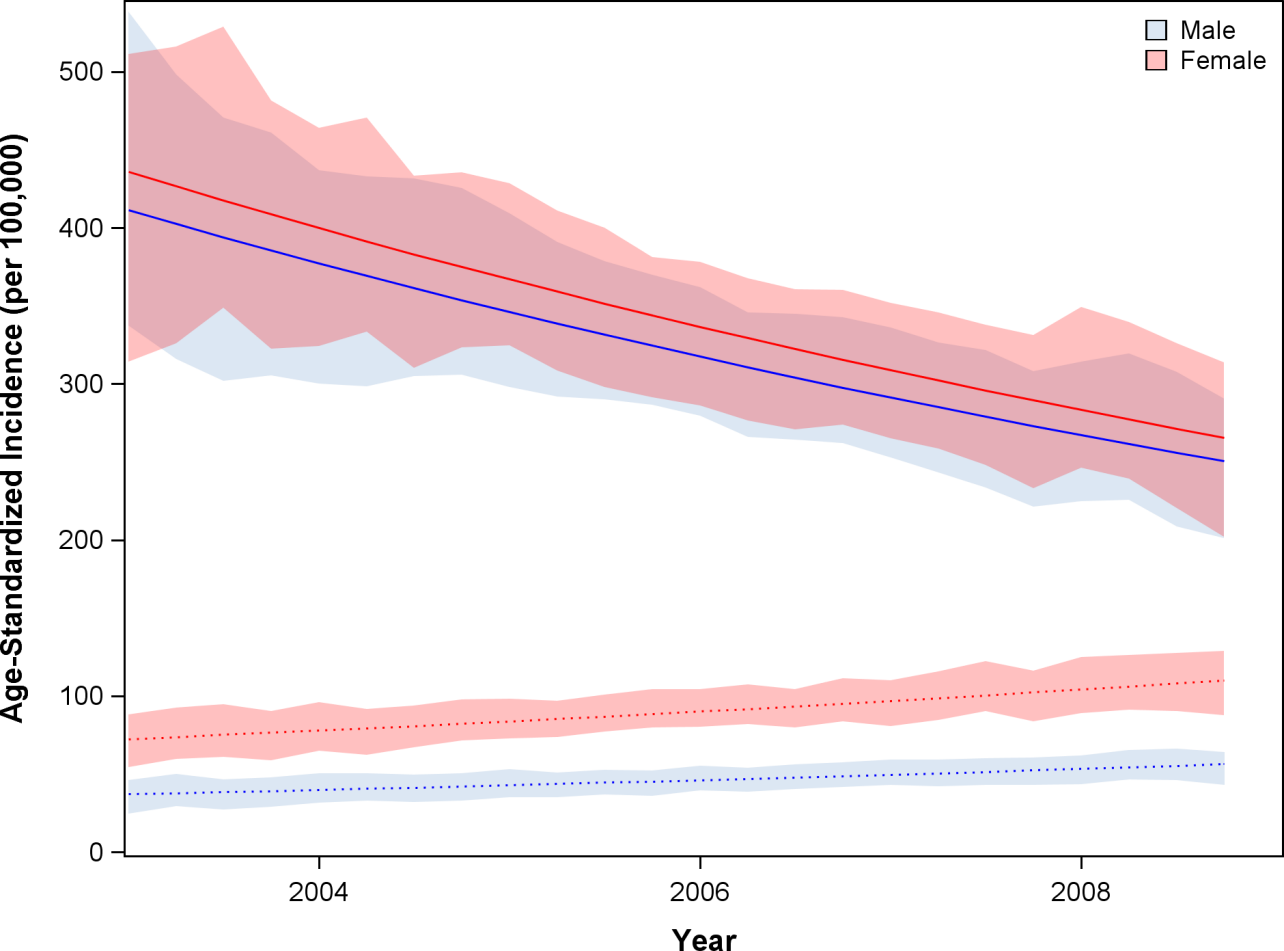
Overall cancer age-adjusted incidence among HIV-infected (solid) and HIV-uninfected (dotted) individuals. Analyses utilized the IPW population.

**Fig 3:**
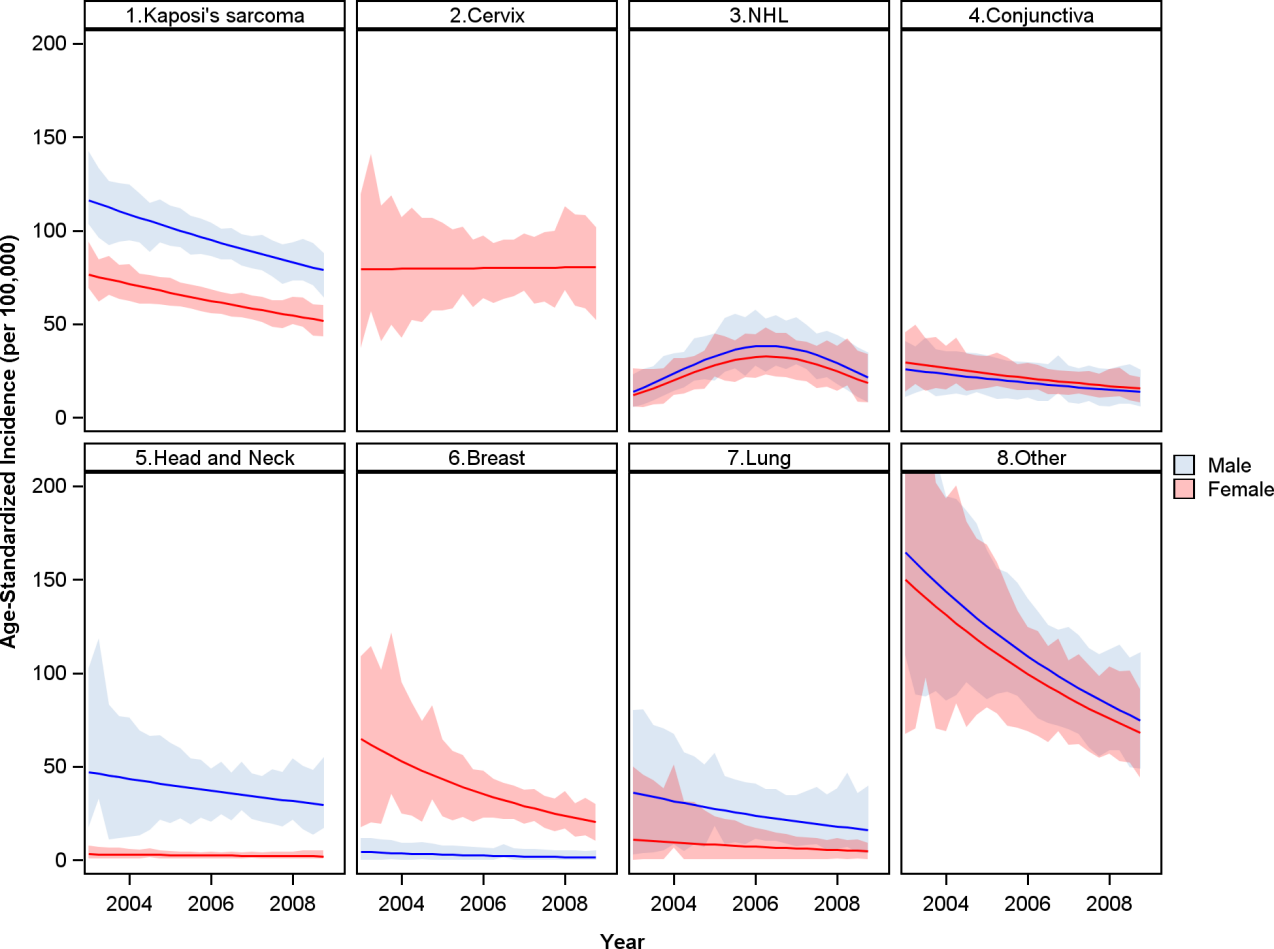
Trend in standardized incidence ratio (SIR) of cancer comparing HIV infected and HIV uninfected populations during ART expansion. Analyses utilized the IPW population. Note: ART, combination antiretroviral therapy.

During ART expansion in Botswana, as estimated in the IPW population, age- and sex-adjusted cancer incidence declined by 8.3% (95% CI -14.1 to -2.1%) per year among HIV-infected individuals (Fig 2). Concurrently, age- and sex-adjusted cancer incidence among individuals without HIV increased by 7.5% (95% CI +1.4 to +15.2%) per year. From 2003 to 2008 the estimated age-standardized incidence ratio (SIR) comparing cancer incidence between the HIV-infected and HIV-uninfected populations fell from 11.1 to 4.4 for men and from 6.0 to 2.4 in for women (Fig 3, p<0.001).

**Fig 4:**
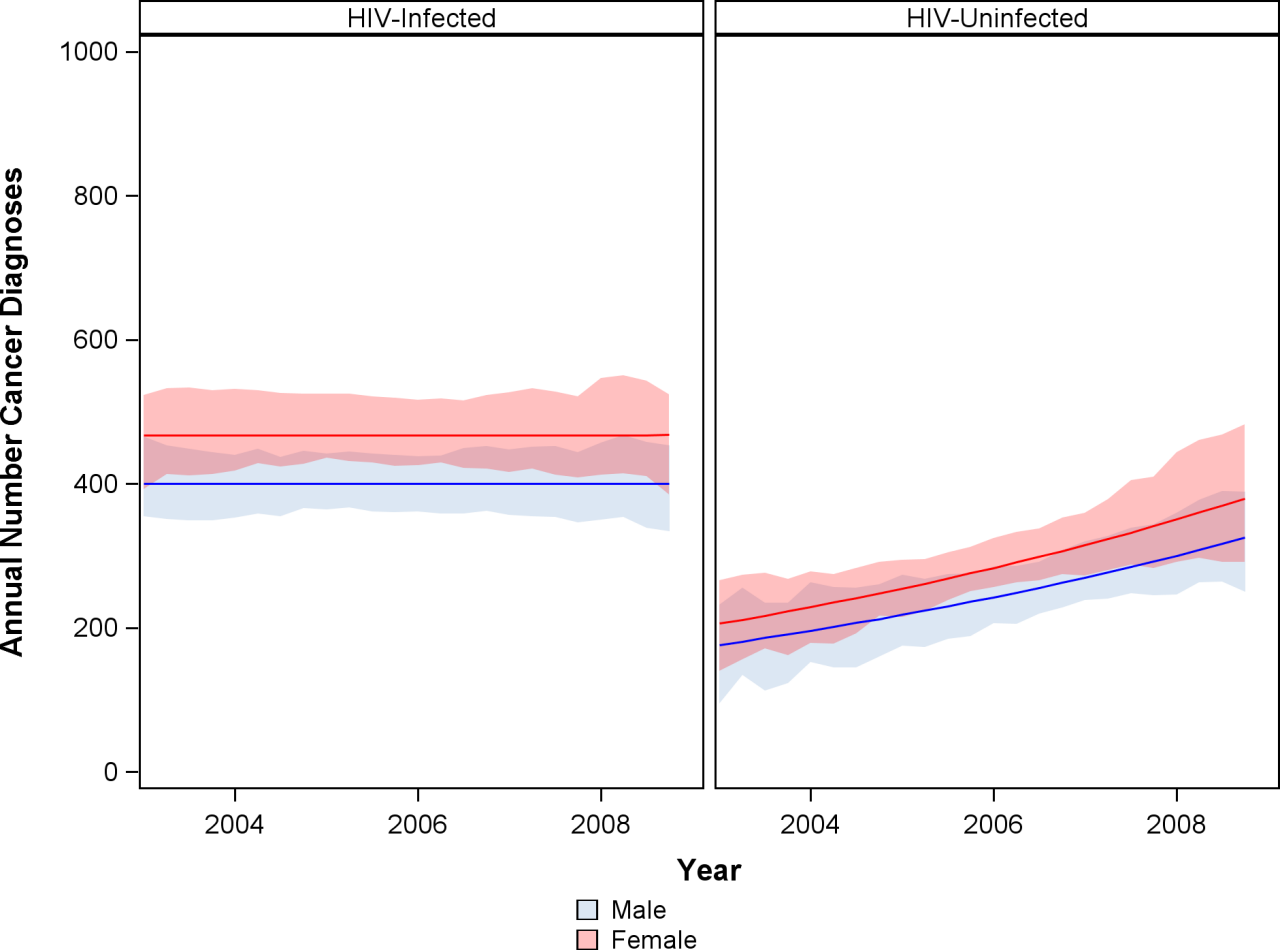
Annual number of cancer diagnoses among HIV-infected and HIV-uninfected in Botswana. Analyses used the IPW population.

However, despite the reduction in incidence, the annual number of new cancers among the growing and aging HIV-infected population remained constant (Fig 4), 0.0% annual change (95% CI -4.3 to +4.6%). During the study period, an estimated 61.7% of incident cancers arose in HIV-infected individuals. HIV infection was attributable for 45.4% of all cancers in men and 36.4% of cancers in women, although expanding availability of ART was associated with decreases in population attributable fraction.

### AIDS-Defining Cancers

**Fig 5:**
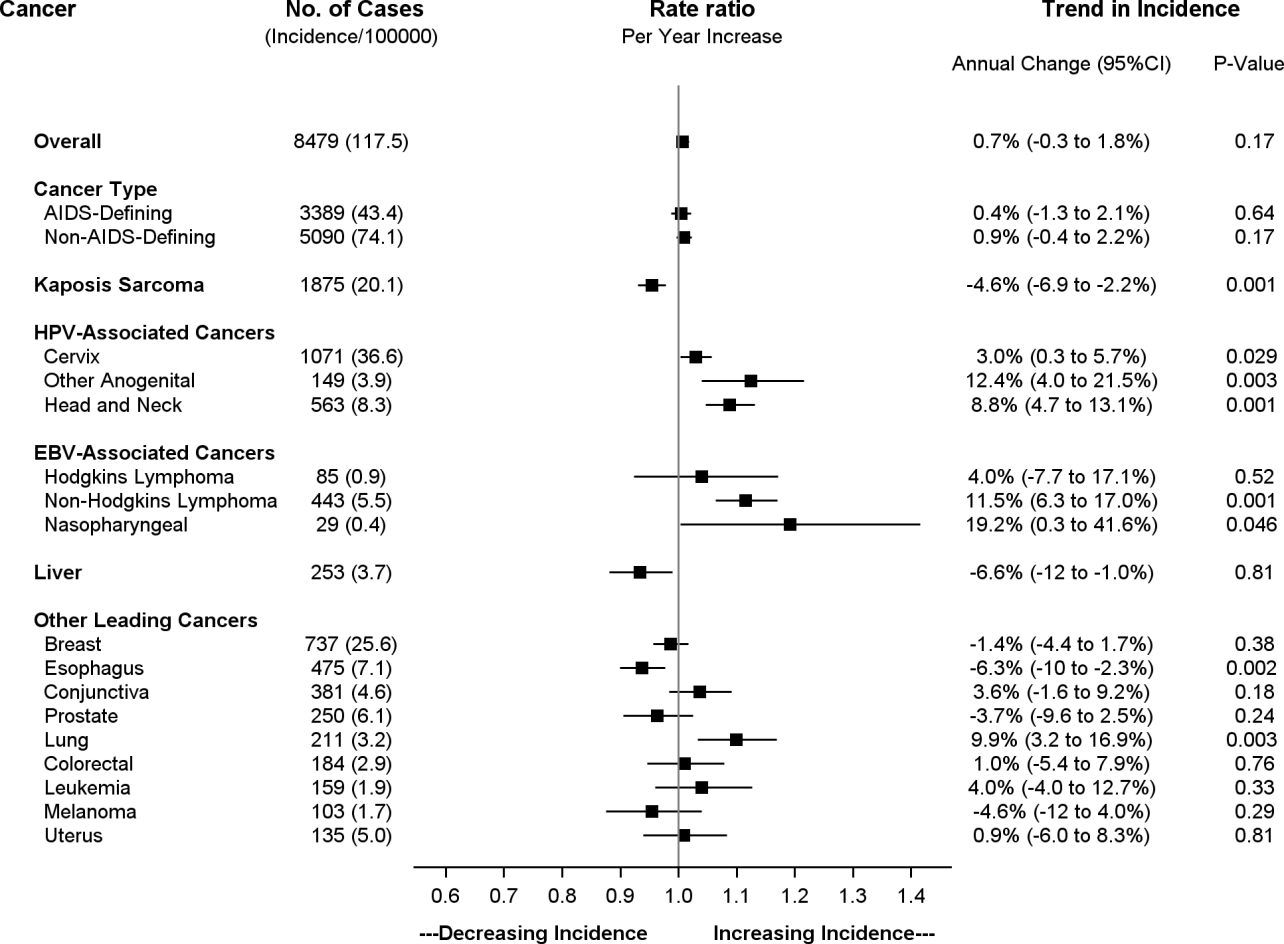
Trends in age-standardized incidence for leading cancers in the overall population (HIV-infected and HIV–uninfected) 2003 to 2008. Incidence estimates for breast and cervical cancer are limited to the female population. Anogenital cancers include squamous cell carcinomas of the vulva, vagina, penis, and anus.

AIDS-defining cancers—Kaposi’s sarcoma, cervical cancer, and non-Hodgkin’s lymphoma—accounted for 40.0% of total cancer cases in the overall population. During the study period there was no significant change in the overall age-standardized incidence of ADC in the overall population, +0.4% annual change (95% CI -1.3 to + 2.1%). However, significant trends were observed in each individual ADC (Fig 5). In the overall population, the incidence of Kaposi’s sarcoma fell by 4.6% annually during the study period (95% CI -6.9 to -2.2). In contrast, the incidence of cervical cancer and non-Hodgkin’s lymphoma both increased during the expansion of ART in the overall population. Incidence of cervical cancer increased by 3.0% annually (95% CI +0.3 to +5.7%) and by 2.8% for every 10% increase in ART coverage (95% CI +0.7 to 4.8%). The increase was greater for non-Hodgkin’s lymphoma with 11.5% annual increase (95% CI +6.3 to +17.0%) and 10.0% increase per 10% increase in ART coverage (95% CI +5.7 to +14.4%).

**Fig 6:**
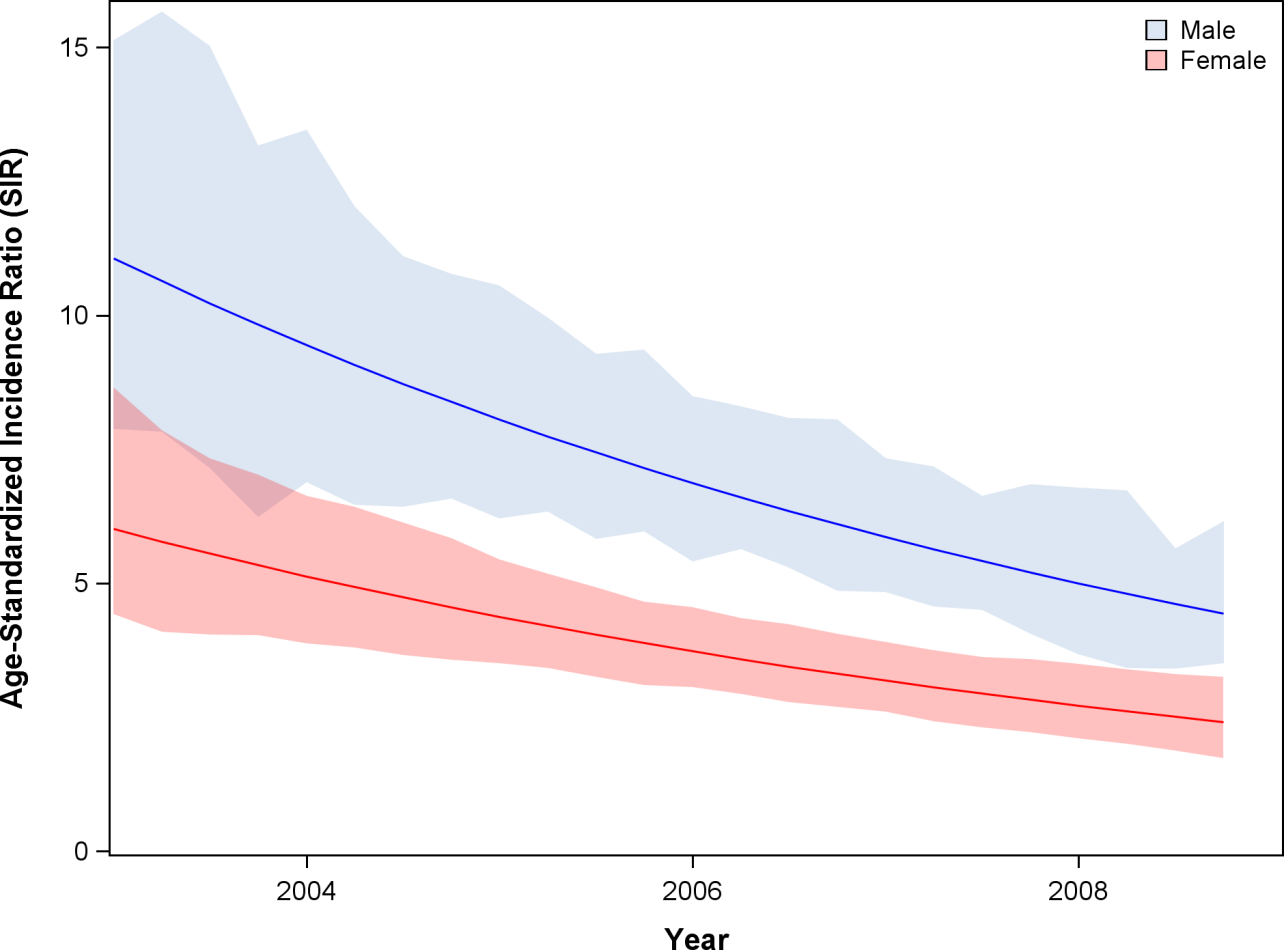
Trends in incidence for leading cancers among HIV-infected population. Estimates from IPW population accounting for changes in overall and age-specific HIV prevalence. Shaded 95% confidence bands from 1000 bootstrap samples. Note: NHL, non-Hodgkin’s lymphoma

In the IPW population accounting for changes in the age structure of HIV-infected individuals, there was also a substantial decrease in Kaposi’s sarcoma incidence among individuals with HIV, 6.5% annually (95% CI -11.1 to -1.6%). However, in these models adjusting for increasing HIV prevalence among older women, cervical cancer incidence was unchanged in the HIV population during the period of ART rollout (0.2% annual change, 95% CI -14.8 to +19.3). Incidence of non-Hodgkin’s lymphoma among HIV-infected individuals significantly increased during the early-ART period and subsequently decreased, p-value for annual and quadratic terms were 0.54 and 0.034, respectively (Table 1 and Fig 6).

**Table 1:** HIV prevalence and change in incidence for leading cancers.

			HIV-Infected		HIV-Uninfected	
	IP Weight median (5 and 95 percentile)	HIV prevalence percent	Annual change Percent (95%CI)	P-value	Annual change Percent (95%CI)	P-value
Kaposi’s sarcoma	0.42 (0.35, 0.51)	99.7%	-6.5% (-11.0 to -1.6%)	0.010	5.5% (-19.7 to 27.0%)	0.78
Non-Hodgkin’s lymphoma	0.91 (0.72, 2.17)	87.6%	—^a^	0.034	2.0 (-27 to 78.7%)	0.63
Cervix	0.96 (0.69, 2.63)	57.5%	-0.2% (-14.8 to 19.3%)	0.99	0.3% (-10.8 to 12.8%)	0.94
Head and Neck	1.50 (0.79, 2.98)	46.1%	-7.7% (-28.3 to 28.0%)	0.68	-1.5 (-19.7 to 27.0%)	0.91
Conjunctiva	1.43 (1.04, 3.32)	91.4%	-10.5% (-24.9 to 3.6%)	0.15	3.7 (-46.8 to 184%)	0.85
Breast	2.13 (1.10, 6.2)	42.8%	-20.8% (-37.9 to 4.9%)	0.14	-2.8 (-15.8 to 11.0%)	0.94
Lung	2.92 (1.38, 6.72)	46.0%	-13.1% (-37.2 to 48.3%)	0.57	25.5 (5.2 to 61.9%)	0.008
Other	2.73 (1.06, 7.25)	39.6%	-12.8% (-24.3 to 1.6%)	0.094	13.5% (3.2 to 27.3%)	0.008
Overall	1.43 (0.38, 5.74)	61.6%	-8.3% (-14.1 to 2.1%)	0.010	7.5% (1.4 to 15.2%)	0.012

Note: IP, inverse probability; 95%CI, 95% confidence interval

^a^ Quadratic term was significant for non-Hodgkin’s lymphoma among HIV-infected individuals—4.5% (95%CI -10.1 to 23.9%) per year and -9.45% (95%CI -19.5 to -1.2%) per year^2^.

### Other Virus-Associated Cancers

Similar to cervical cancer, incidence of other cancers linked to human papilloma virus (HPV) increased in the overall population, including head and neck cancers (+8.8% annually, 95% CI +4.7 and +13.1%) and other anogenital cancers (+12.4% annually, 95% CI +4.0 to 21.5%). Overall, all cancers associated with HPV (including cervical cancer) increased 3.9% annually (95% CI +1.4 to +6.5%) and 3.6% per increase of ART coverage by 10% (95% CI +1.6 to +5.7%) in the overall population. By the end of the surveillance period, HPV-associated cancers accounted for 27.2% of all malignancies in Botswana, increasing from 19.6% during the initial implementation of the ART program.

Observed increases in HPV-associated cancers in the total population may reflect an aging and expanding HIV population. In analyses in the IPW population adjusting for these demographic shifts, no change with ART expansion was detected for HPV-associated cancers among HIV-infected individuals (-1.2% annually, -11.9 to +12.3%, data not shown).

In the overall population, cancers associated with Epstein Barr virus (EBV)—NHL, Hodgkin’s lymphoma, and nasopharyngeal cancer—increased in incidence during expansion of ART, 11.0% annually (95% CI +6.3 to +15.8%) and 9.7% per 10% increase in ART coverage (95% CI +5.9% to +13.7%). However, trends in individual EBV-associated cancers were difficult to evaluate due to low frequencies. Incidence of Hodgkin’s lymphoma appeared stable (+4.0% annually, 95% CI -7.7 to +17.1%) while that of nasopharyngeal carcinoma increased (+19.2% annually, 95% CI +0.3 to +41.6%). Using the IPW population, no significant change in incidence was observed in EBV-associated cancers in HIV-infected individuals (+5.3 annually, 95% CI -6.5 to 22.2%, data not shown).

Incidence of liver cancer, likely related to prevalent chronic hepatitis B (HBV) infection in Botswana,[27] appeared to decline in the overall population, -6.6% annually (95% CI -12.0 to -1.0%). This trend was more prominent among individuals with HIV in the IPW population, although the decline was not statistically significant (-43.4% annually, 95% CI -66.6% to +2.3%, data not shown). During this period there was not a dedicated HBV treatment program, but nearly all ART regimens included at least one HBV-active agent.

### Cancers Not Associated with Viral Infection

Non-AIDS-defining cancers without an established infectious link had relatively stable incidence during the period of rapid ART expansion in Botswana. In the overall population, we observed increasing incidence of lung cancer (+9.9% annually, 95% CI +3.2 to 16.9%) that may be related to increasing tobacco use.[28] We also observed decreasing incidence of esophageal cancer (-6.3% annually, 95% CI -10.0 to -2.3%).

In the IPW population, breast and lung cancer were the most common incident cancers not associated with viral infection in HIV-infected individuals. There was a non-significant decreasing trend in incidence for breast cancer (-20.8 annually, 95% CI -379.9 to 4.9%) from 2003 to 2008. Overall, incidence of non-viral associated cancers declined by 8.3% annually (95% CI -14.1 to -2.1%, data not shown) among the HIV-infected population and increased by 7.5% annually (95% CI +1.4 to 15.2%, data not shown) among the HIV-uninfected population.

## Discussion

Through analysis of individual cases from the BNCR, we have found that cancer risk, particularly for Kaposi’s sarcoma, decreased among HIV-infected individuals with establishment of a comprehensive ART program with high coverage. However, with an enlarging and aging HIV-infected population in Botswana, the number of cancers did not decline with ART expansion. The incidence of HPV-associated cancers and non-Hodgkin’s lymphoma increased 2-fold from 2003 to 2008. Cervical and other HPV-associated cancers, mostly arising in HIV-infected persons, accounted for over 25% of all tumors following ART expansion. Cancers that are increasing in incidence are associated with high mortality and treatment costs, adding considerably to the overall burden of an intense HIV epidemic.

In the US, ART was associated with sharp declines in both Kaposi’s and non-Hodgkin’s lymphoma.[2] Findings from this analysis demonstrate a contrasting trajectory. Similar to findings elsewhere in Africa,[6, 7] Botswana has had only a modest decrease in KS incidence and persistent heavy burden of other HIV-associated cancers despite remarkable improvements in ART access. In part, the apparently discrepant trends are likely related to challenges in securing a diagnosis in a constrained health system, particularly early in the ART program when AIDS deaths were high. Additionally, obtaining a cancer diagnosis is frequently a long process and patients with competing opportunistic conditions may not survive to complete it. This could explain the initial rise in NHL incidence with ART in Botswana. Differences in competing risks between high- and low and middle-income countries may explain some of the observed differences in cancer trends with ART.

The predominance of HPV-associated cancers suggests that a principal reason for increasing burden of HIV-associated cancers is the majority female HIV epidemic in the region and the high prevalence of oncogenic viral infections. While ART limits the duration and frequency of cervical HPV infections,[29] it does not appear to reliably arrest tumor development once initiated.[30] Consequently, the burden of cervical cancer and other HPV-associated cancers may have increased due to improved HIV afforded by ART. Vaccination and screening for HPV or dysplasia are important responses, but these initiatives will not abate the need for comprehensive oncologic care for the rising wave of cervical, penile, vulvar, and anal cancers over the next decade.

While our analysis utilized data from a national cancer registry with high levels of case capture, a number of limitations are important to consider when interpreting these results. Most importantly, HIV status is incompletely captured and the use of weights to mitigate biased ascertainment of HIV status increased the variance of estimates. As a result, power in the IPW population analyses was reduced to detect changes in incidence for individual cancers. Additionally, while the IPW population had similar HIV prevalence to a reference hospital cohort providing reassurance against bias, residual misclassification could bias trends. However, the unmeasured factor would need to be has influential as the strongest predictors of censoring of HIV status (cancer type and year of diagnosis) to modify estimates by more than 5%. The registry most efficiently captures pathologically confirmed cases and those that present for specialized oncologic management, and as a result likely underrepresents incidence of Kaposi’s sarcoma, cancers arising in sites difficult to biopsy (e.g. brain, lung, or liver), and asymptomatic cancers. Similarly, case capture by the registry is vulnerable to changes in clinical and diagnostic capacity, irrespective of the ART program. We did not detect significant changes in incidence over time for selected cancers not expected to change with ART expansion, arguing against ascertainment bias; however we cannot exclude the possibility that improved health system function (as evidenced by decreased death certificate only cases) could have contributed to the observed increases in some cancers.

In conclusion, increasing ART coverage in Botswana was associated with decreased age-adjusted incidence of cancer among HIV-infected individuals, largely due to drop in Kaposi’s sarcoma. However, the overall number of cancers was unchanged in the enlarging and aging HIV-infected population. Despite high coverage of ART, the majority of tumors continue to arise in HIV-infected individuals. In addition to substantial morbidity and mortality, the increased cancer burden has placed considerable additional strain on the health system already managing an intense HIV epidemic. With the first comprehensive ART program in Africa, trends in cancer incidence in Botswana may reflect the emerging cancer epidemic in the region fueled by chronic HIV infection. Unfortunately, expansion of ART for individuals with low CD4 counts alone is unlikely to substantially reduce cancer burden in sub-Saharan Africa.
