# Correction: Cancer Incidence following Expansion of HIV Treatment in Botswana

**DOI:** 10.1371/journal.pone.0138742

**Published:** 2015-09-16

**Authors:** Scott Dryden-Peterson, Heluf Medhin, Malebogo Kebabonye-Pusoentsi, George R. Seage, Gita Suneja, Mukendi K. A. Kayembe, Mompati Mmalane, Timothy Rebbeck, Jennifer R. Rider, Myron Essex, Shahin Lockman

The images for Figs 3 and 6 are incorrectly switched. The image that appears as Fig 3 should be Fig 6, and the image that appears as Fig 6 should be Fig 3. The figure captions appear in the correct order. Please see the corrected figures here.

**Fig 3:**
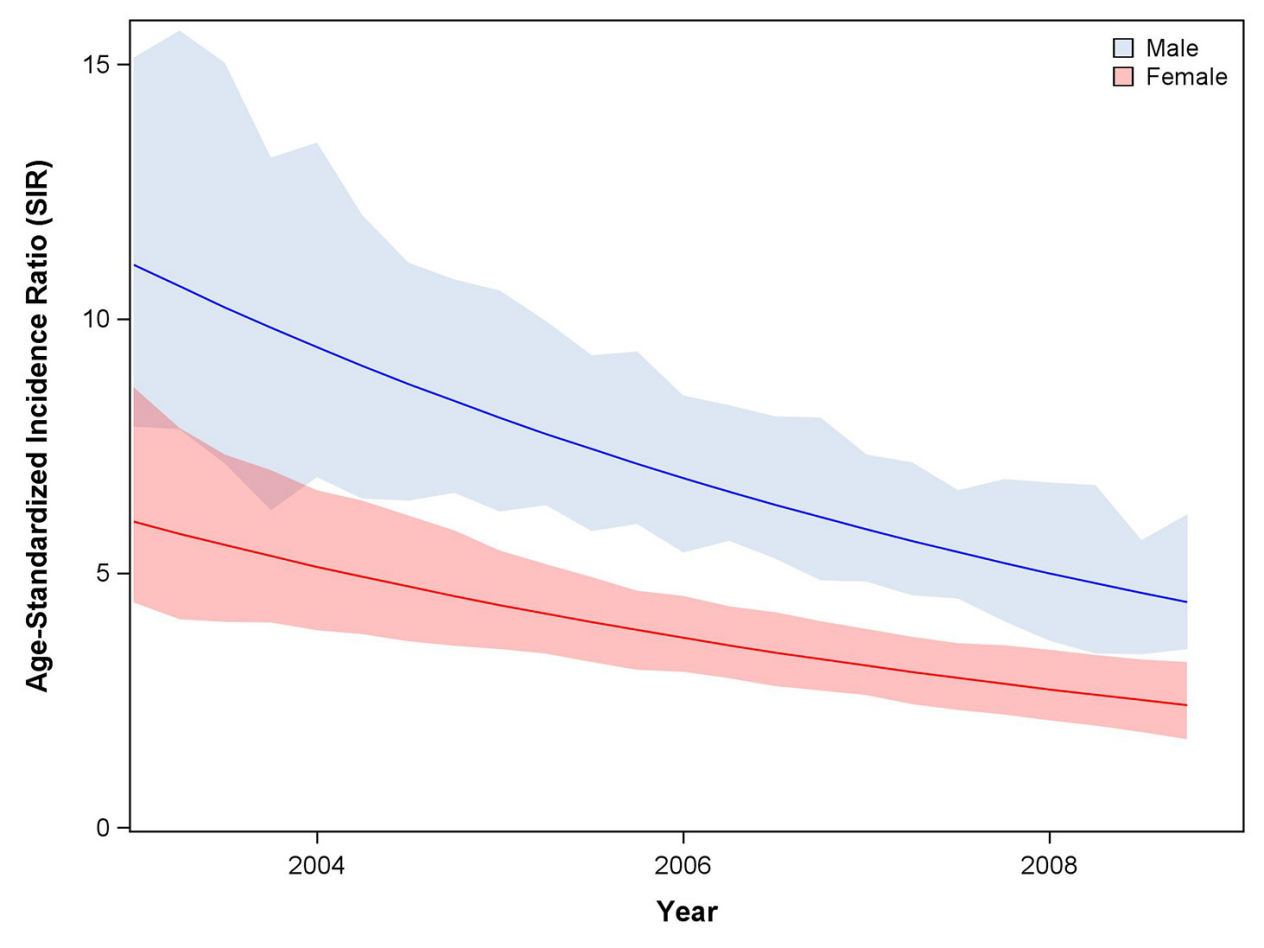
Trend in standardized incidence ratio (SIR) of cancer comparing HIV infected and HIV uninfected populations during ART expansion. Analyses utilized the IPW population. Note: ART, combination antiretroviral therapy.

**Fig 6:**
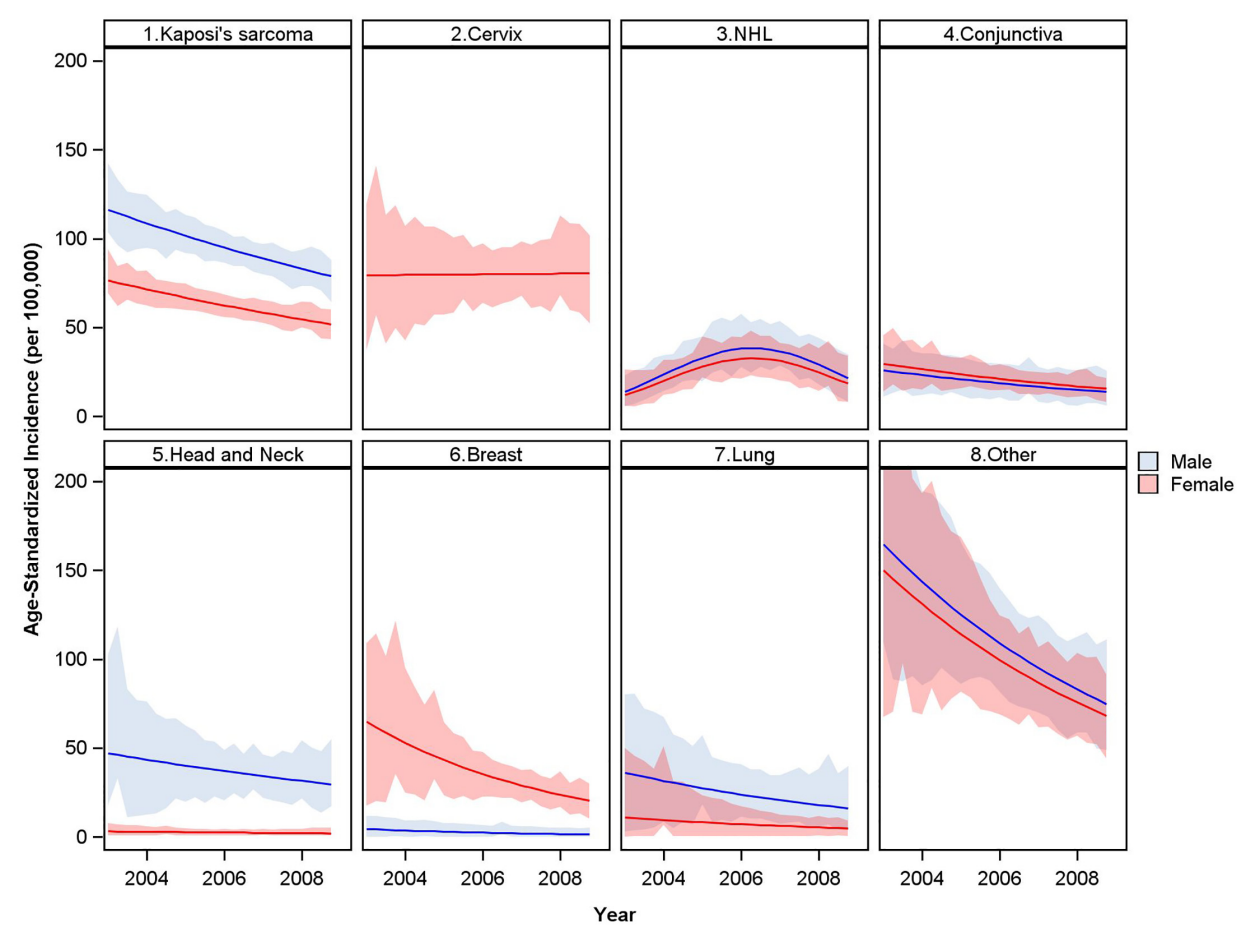
Trends in incidence for leading cancers among HIV-infected population. Estimates from IPW population accounting for changes in overall and age-specific HIV prevalence. Shaded 95% confidence bands from 1000 bootstrap samples. Note: NHL, non-Hodgkin’s lymphoma
